# Renal Failure in Lithium-Treated Bipolar Disorder: A Retrospective Cohort Study

**DOI:** 10.1371/journal.pone.0090169

**Published:** 2014-03-26

**Authors:** Helen Close, Joe Reilly, James M. Mason, Mukesh Kripalani, Douglas Wilson, John Main, A. Pali S. Hungin

**Affiliations:** 1 Durham Clinical Trials Unit, School of Medicine, Pharmacy and Health, Durham University, Queen’s Campus, Wolfson Research Institute, Stockton-on-Tees, United Kingdom; 2 School of Medicine, Pharmacy and Health, Durham University, Queen’s Campus, Wolfson Research Institute, Stockton-on-Tees, United Kingdom; 3 Tees, Esk & Wear Valleys NHS Foundation Trust, West Park Hospital, Darlington, County Durham, United Kingdom; 4 South Tees Hospitals NHS Foundation Trust, Middlesbrough, United Kingdom; Maastricht University Medical Centre, Netherlands

## Abstract

**Objective:**

Lithium users are offered routine renal monitoring but few studies have quantified the risk to renal health. The aim of this study was to assess the association between use of lithium carbonate and incidence of renal failure in patients with bipolar disorder.

**Methods:**

This was a retrospective cohort study using the General Practice Research Database (GPRD) and a nested validation study of lithium exposure and renal failure. A cohort of 6360 participants aged over 18 years had a first recorded diagnosis of bipolar disorder between January 1, 1990 and December 31, 2007. Data were examined from electronic primary care records from 418 general practices across the UK. The primary outcome was the hazard ratio for renal failure in participants exposed to lithium carbonate as compared with non-users of lithium, adjusting for age, gender, co-morbidities, and poly-pharmacy.

**Results:**

Ever use of lithium was associated with a hazard ratio for renal failure of 2.5 (95% confidence interval 1.6 to 4.0) adjusted for known renal risk factors. Absolute risk was age dependent, with patients of 50 years or older at particular risk of renal failure: Number Needed to Harm (NNH) was 44 (21 to 150).

**Conclusions:**

Lithium is associated with an increased risk of renal failure, particularly among the older age group. The absolute risk of renal failure associated with lithium use remains small.

## Introduction

Lithium carbonate has been a cornerstone of treatment of bipolar disorder for more than 40 years and more recently a key augmentive treatment for unipolar depression. Its narrow therapeutic window and potential for lethal toxicity [1] has led to an increasing use of alternative agents, particularly valproate. The effect of lithium on the kidney influences prescribing decisions, and monitoring of renal function is part of standard care. Uncertainty remains about the level of renal harm associated with lithium use for bipolar treatment, as well as the role of duration of exposure [2]. Its known effect on tubular function (resulting in nephrogenic diabetes insipidus) has been contrasted with a less well-established association with renal failure, possibly confounded by other known risk factors [3]. Reported epidemiological studies are either cross-sectional, small cohort [4]–[12], or small case-control [13]–[19]. Of the case-control studies none were adequately powered to analyse the effect of age or concomitant drugs [13]–[19]. A recent systematic review [20] demonstrated that the evidence base for renal monitoring for patients taking lithium is surprisingly weak. No large-scale study has been conducted with adequate power to detect renal failure outcomes in lithium use, adjusted for other risk factors. However, lithium prescribing remains widespread, with evidence of anti-suicidal effects [21], [22] and a clear role within clinical guidelines for bipolar disorder [23], [24].

Because of the availability of a large, UK-representative study population, we were able to estimate the incidence of renal outcomes in the General Practice Research Database (GPRD, http://www.gprd.com/) cohort during the period 1990–2007 in patients with bipolar disorder according to age, gender, and use of lithium. This study estimates the relative hazard of renal failure (the primary outcome) and renal impairment (the secondary outcome) in patients with bipolar disorder according to lithium use and adjusted for age, gender, known risk factors for renal disease and lithium exposure over time. The GPRD has been extensively validated to record primary care prescriptions; an unknown proportion of lithium prescribing may originate from secondary care, thus the study also sought to validate GPRD renal failure, ever-use of lithium and lithium exposure records.

## Methods

### Ethics Statement

Data was accessed within limits set out by the Medical Research Council licence agreement for academic access with Medical Research Ethics Committee ethical approval. The proposal was approved by the Independent Scientific Advisory Committee of the GPRD (protocol number 07_107R).

### Data Source

A retrospective cohort study was conducted, accessing records from the GPRD, which provided anonymised access to electronic primary care medical records and prescriptions for approximately 6% of the UK population. The age and sex distribution of the GPRD population was similar to the general population and each contributing GP practice was audited monthly to ensure data quality. The database contained information on consultation dates, diagnosis, symptoms, procedures or investigation, referrals, their outcome, drug frequency and dose. Diagnoses and prescriptions were recorded using OXMIS and Prescription Pricing Authority (PPA) codes respectively; all codes for bipolar disorder, renal outcomes, medications and all covariates are available on request from the corresponding author. Data were accessed from the GPRD under the MRC licence for academic groups [25].

### Disease Definitions

Renal disease occurring in patients was defined using two definitions capturing severity and spectrum of disease. The primary outcome, renal failure (RF), included diagnostic and referral codes indicating end stage renal failure, chronic kidney disease stages 4 or 5 (CKD4 and CKD5) and renal replacement therapy (dialysis or transplantation). The secondary outcome, renal impairment (RI) included diagnostic codes for milder degrees of renal dysfunction including chronic kidney disease stage 3 (CKD3). Disease definitions were developed under the supervision of a consultant nephrologist (JM).

### Study Population

Patients were identified for inclusion on the basis of diagnosis of bipolar disorder recorded in the medical record. General practices were selected that provided quality-checked data from 1st January 1990–31st December 2007, including 2,130,070 patients registered with these practices aged 18 years or over. Patients with less than one year of GP registration were excluded, as were patients with renal cancer, congenital abnormalities or renal conditions relating to pregnancy. Male and female patients were identified with a first recorded occurrence of bipolar disorder after 1 January 1990.

Patient records were examined for first and subsequent prescriptions of lithium. In addition to renal outcomes, patient records were evaluated for diagnoses of diabetes mellitus, cardiovascular disease, cerebrovascular disease, liver disorder, other diseases of the urinary system (renal sclerosis, small kidney); prescriptions for nonsteroidal anti-inflammatory drugs (NSAIDs), paracetamol, angiotensin-converting enzyme (ACE) inhibitors, diuretics, β-adrenoceptor-blocking drugs and other antihypertensives, antipsychotics (quetiapine, risperidone, or olanzapine), and mood stabilizing drugs (sodium valproate, lamotrigine, or carbamazapine). Reflecting variable reporting, smoking and alcohol status were crudely categorized by “never” or “ever” usage. Mean BMI was calculated using all BMI readings within the study window and categorized as underweight (<18.5), normal (18.5 to 24.9), overweight (25 to 29.9), obese (30 to 39.9), morbidly obese (>40). GP practices were allocated a quintile score for socioeconomic status based in the Index of Multiple Deprivation (IMD) [26].

### Drug Exposure Definitions

Lithium use incorporated the following proprietary and generic classifications: Camcolit, Liskonum, Priadel, Li-Liquid, lithium carbonate and lithium citrate. A pre-specified definition of lithium exposure duration adjusted using the Defined Daily Dose method [27] was planned, but following a validation exercise (see below and results), dose-adjusted exposure duration was not included in the analyses presented.

Exposure to NSAIDs was categorized as non-use, ≤30 days (short-term use) and >30 days (long-term use). Cardiovascular dose aspirin was excluded from NSAID categorisation. Other drugs recorded and available to statistical models included β-adrenoreceptor blocking drugs, diuretics, ace inhibiting drugs, antipsychotics (quetiapine, risperidone, or olanzapine), and mood stabilizing drugs (sodium valproate, lamotrigine, or carbamazapine). Exposure was defined as at least one prescription record in the time window following bipolar diagnosis and before a renal outcome (if occurring).

### Validation

A validation exercise was conducted with permission from GPRD which aimed to examine (i) the primary outcome of renal failure, (ii) ever-use of lithium in those with a record of renal failure, and (iii) lithium exposure duration in those with a record of renal failure. We requested access to the full text of general practice records covering the duration within the cohort for each case. Validation was conducted by sending a questionnaire to registered GPs asking for confirmation and additional details of renal failure (and bipolar disorder) diagnosis, lithium exposure, date of birth and gender. Copies of confirmatory patient notes were requested.

### Analysis

The relative risks of renal outcomes (RI and RF) were estimated as hazard ratios (HRs) for lithium use compared to non-use, using Cox proportional hazard models. Models were subsequently adjusted for patient demographics, comorbidity and concomitant drug use. These included baseline variables (recorded at the time of diagnosis of bipolar disorder), and emergent variables (recorded following diagnosis of bipolar disorder but before diagnosis of a renal outcome) putatively related to renal disease.

We carried out both forward and backward stepwise covariate selection within the Cox proportional hazard model. All covariates (age, gender, alcohol use, smoking, BMI, GP practice Index of Multiple Deprivation (IMD), cerebrovascular disease, cardiovascular disease, liver failure, diabetes, malignancy, hypertension, atypical antipsychotics, mood stabilizers, beta-blockers, diuretics, ace-inhibitors, paracetamol, NSAIDS) were initially included with entry testing based on the significance of the score statistic, and removal testing based on the probability of a likelihood-ratio statistic based on conditional parameter estimates. Final selected models were re-estimated to provide unconditional estimates. Data management was generally performed using Stata 10IC [28] and the Cox regression proportional hazard computations [29] and Kaplan-Meier survival analysis used SPSS [30]. Final models were tested for (and rejected) time-dependency and interactions between fitted variables. Precision of estimates is reported using 95% confidence intervals.

## Results

### Baseline Cohort Characteristics

**Figure 1:**
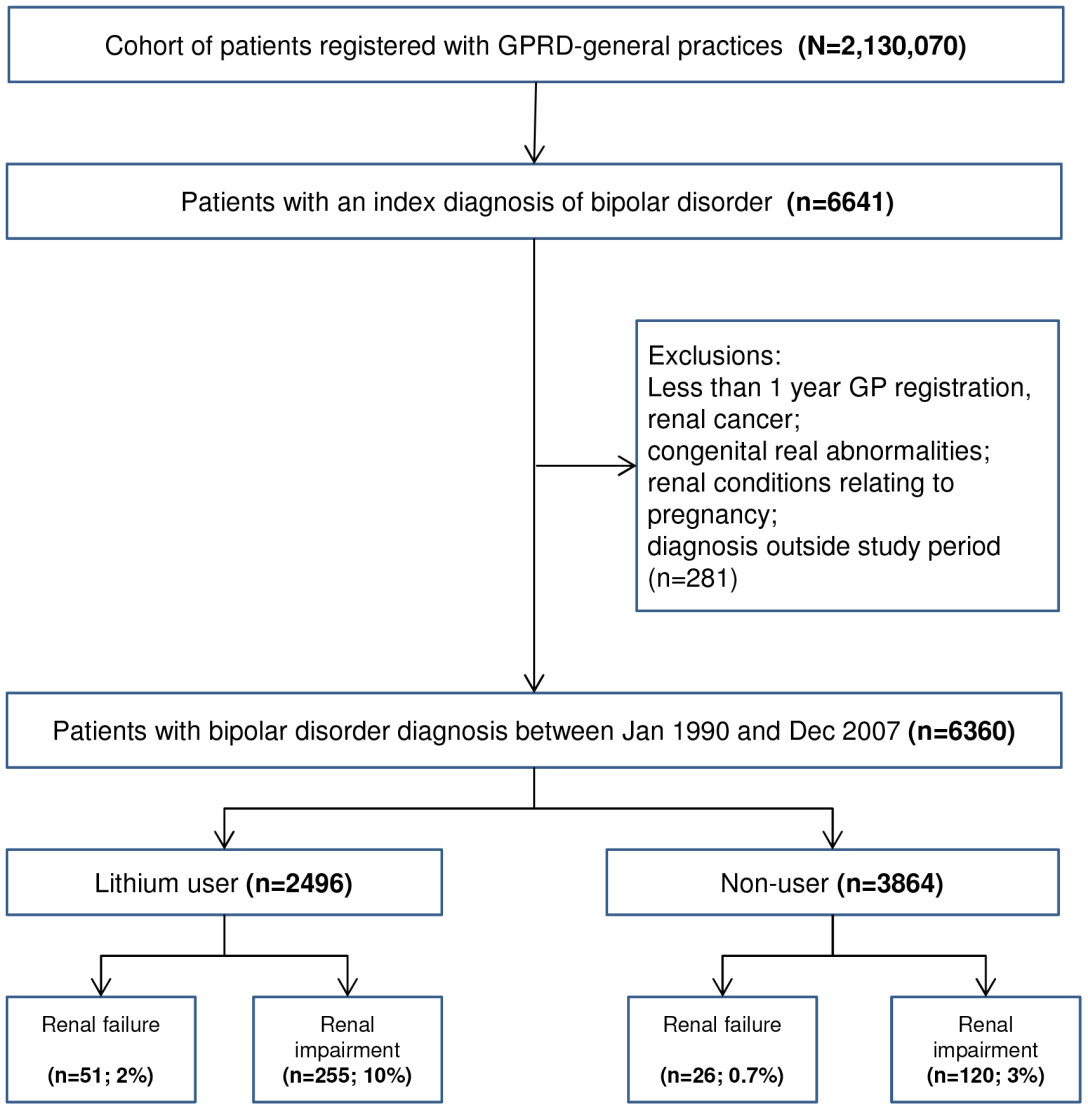
Flowchart of study participants.

The cohort consisted of 6360 patients within 418 practices with a first diagnosis of bipolar disorder recorded from 1st January 1990 to 31st December 2007 (figure 1). The cohort mean age at diagnosis was 48.8 years (range 18 to 105), 60.5% were women and median follow-up was 5.4 years (3 months –18 years, SD 4.6 years). A total of 22% of patients had ten or more years of follow-up. All cause mortality during follow-up was 773 (12.2%). BMI, smoking status and alcohol use were inconsistently recorded (records in 54.9%, 93.3% and 84.3% respectively) and status varied over time. The socio-economic status (SES) of practices was evenly spread across the cohort from least to most deprived, with no clinically important differences in lithium prescribing rates.

### Emergent Cohort Characteristics

Within the cohort, following diagnosis of bipolar disorder but before any diagnosis of renal disease a range of emergent conditions were diagnosed (Table 1). Most variables were not associated with renal disease within regression models, or only fitted in univariate analysis. A number of these variables were correlated: diabetes, hypertension and use of diuretics, beta-blockers and ACE inhibitors and thus not independently explanatory.

**Table 1:** Cohort baseline and emergent characteristics at diagnosis of bipolar disorder, according to use or nonuse of lithium.

		Total N = 6360 (%)	Lithium Users N = 2496 (%)	Non-Users N = 3864 (%)	P^5^
**Age (y)**	18 to 34	1482 (23)	473 (19)	1009 (26)	
	35 to 49	1983 (31)	811 (33)	1172 (30)	
	50 to 64	1626 (26)	703 (28)	923 (24)	
	65 to 79	960 (15)	402 (16)	558 (14)	
	80+	309 (4.9)	107 (4.3)	202 (5.2)	
	Mean (SD)	48.8 (17)	49.8 (16)	48.1 (18)	<0.001
**Gender**	Male	2511 (40)	995 (40)	1516 (39)	0.59
	Female	3849 (61)	1501 (60)	2348 (61)	
**Alcohol user** 1		4470 (83)^2^	1846 (85)	2624 (82)	0.001
**Smoker** 1		3649 (61)^3^	1430 (60)	2219 (62)	0.16
**BMI** 1	Underweight	78. (2.2)	23 (1.5)	55 (2.8)	
	Normal	1094 (31)	472 (31)	622 (32)	
	Overweight	1202 (34)	533 (35)	669 (34)	
	Obese	990 (28)	439 (29)	551 (28)	
	Morbidly Obese	127 (3.6)^4^	62 (4.1)	65 (3.4)	
	Mean (SD)	28. (6.3)	28 (6.1)	27.9 (6.4)	<0.001
**Practice IMD** 6	0	1272 (20)	506 (20)	766 (20)	0.07
	1	1005 (16)	430 (17)	575 (15)	
	2	1229 (20)	495 (20)	734 (19)	
	3	1385 (22)	485 (20)	900 (23)	
	4	1469 (23)	580 (23)	889 (23)	
**Comorbidity (any)**		1410 (22)	549 (22)	861 (22)	0.76
	Cerebrovascular Disease	286 (4.5)	76 (3.0)	210 (5.4)	<0.001
	Cardiovascular Disease	765 (12)	282 (11)	483 (13)	0.16
	Liver Failure	14 (0.2)	5 (0.2)	9 (0.2)	0.79
	Diabetes	492 (7.7)	205 (8.2)	286 (7.4)	0.25
	Malignancy	197 (3.1)	93 (3.7)	104 (2.7)	0.02
	Hypertension	638 (10)	276 (11)	326 (8.4)	0.001
**Atypical antipsychotics**		1977 (31)	868 (35)	1109 (29)	<0.001
**Mood stabilizers**		1656 (26)	670 (27)	986 (26)	0.28
**Beta-blockers**		912 (14)	434 (17)	478 (12)	<0.001
**Diuretics**		1192 (19)	452 (18)	740 (20)	0.28
**Ace-inhibitors**		645 (10)	253 (10)	392 (10)	0.94
**Paracetamol**		2648 (42)	1218 (49)	1430 (37)	<0.001
**NSAIDS**.	Long-term (>30d)	202 (3.2)	76 (3.0)	126 (3.3%)	0.001

1 Earliest recorded value following diagnosis, as a proportion of those in whom status is recorded.

2 15.7% of patients had missing data.

3 6.1% of patients had missing data.

4 45.1% of patients had missing data.

5 p-values: comparison of binary variables by adjusted χ2 test; continuous variables by Student’s t-test; multiple category variables by χ2 test adjusted for trend.

6 Index of Multiple Deprivation (IMD) based on practice post-code. 0 is least deprived, 4 is most deprived.

### Lithium Exposure

A total of 2496 patients (39% of the cohort) were prescribed at least one prescription of at least 30 days duration for lithium. The mean age of commencing lithium therapy was 49.8 years (SD 16 y, range 17–97 y). Lithium users were recruited at a similar rate to non-lithium users over the period. Users and non-users of lithium had clinically similar baseline demographic characteristics (Table 1), although small differences appear statistically significant in this large cohort. Lithium users were slightly older than non-lithium users at recruitment (49.8 vs. 48.1 years), and had longer mean duration of follow-up (7.4 vs. 5.7 years).

### Renal Outcomes

During cohort follow-up, there were 77 cases of incident renal failure (RF) and 375 incident cases of renal impairment (RI). The unadjusted prevalence of RF (2.0% vs. 0.7%) and RI (10.2% vs. 3.1%) was higher in lithium users than non-users (Table 2).

**Table 2:** Unadjusted and adjusted hazard ratios for renal failure and renal impairment among lithium users and non-users.

	n/N (%)	HR (95%CI), p Unadjusted	HR (95%CI), p Age and sex-adjusted
**Renal Failure**	77/6360 (1.2)		
Lithium use (unvalidated)	49/2494 (2.0)	2.1 (1.3 to 3.4), 0.002	2.2 (1.4 to 3.6), 0.001
Non-use	28/3866 (0.7)		
Lithium use (validated)	51/2496 (2.0)	2.4 (1.5 to 3.8), <0.001	2.5 (1.6 to 4.0), <0.001
Non-use	26/3864 (0.7)		
**Renal Impairment**	375/6360 (5.9)		
Lithium use	255/2494 (10.2)	2.5 (2.0 to 3.1), <0.001	2.7 (2.2 to 3.4), <0.001
Non-use	120/3866 (3.1)		

Adjusted for age and gender, ever-use of lithium was associated with an increased risk of developing renal failure (HR: 2.5 (95% confidence interval 1.6 to 4.0)) and renal impairment (HR: 2.7 (2.2 to 3.4)). Several predictor variables showed a very weak but significant bivariate correlation with renal failure but did not fit in fully adjusted models, namely: ACE-inhibitors (Pearson correlation coefficient *r* = 0.082, p<0.001), beta-blockers (Pearson correlation coefficient *r* = 0.041, p = 0.001), hypertension (Pearson correlation coefficient *r* = 0.053, p = 0.001), and paracetamol (Pearson correlation coefficient *r* = 0.049, p = 0.001). The following covariates did not show a bivariate correlation with renal failure; age, gender, alcohol use, smoking, BMI, GP practice Index of Multiple Deprivation (IMD), cerebrovascular disease, cardiovascular disease, liver failure, malignancy, atypical antipsychotics and mood stabilizers.

**Figure 2:**
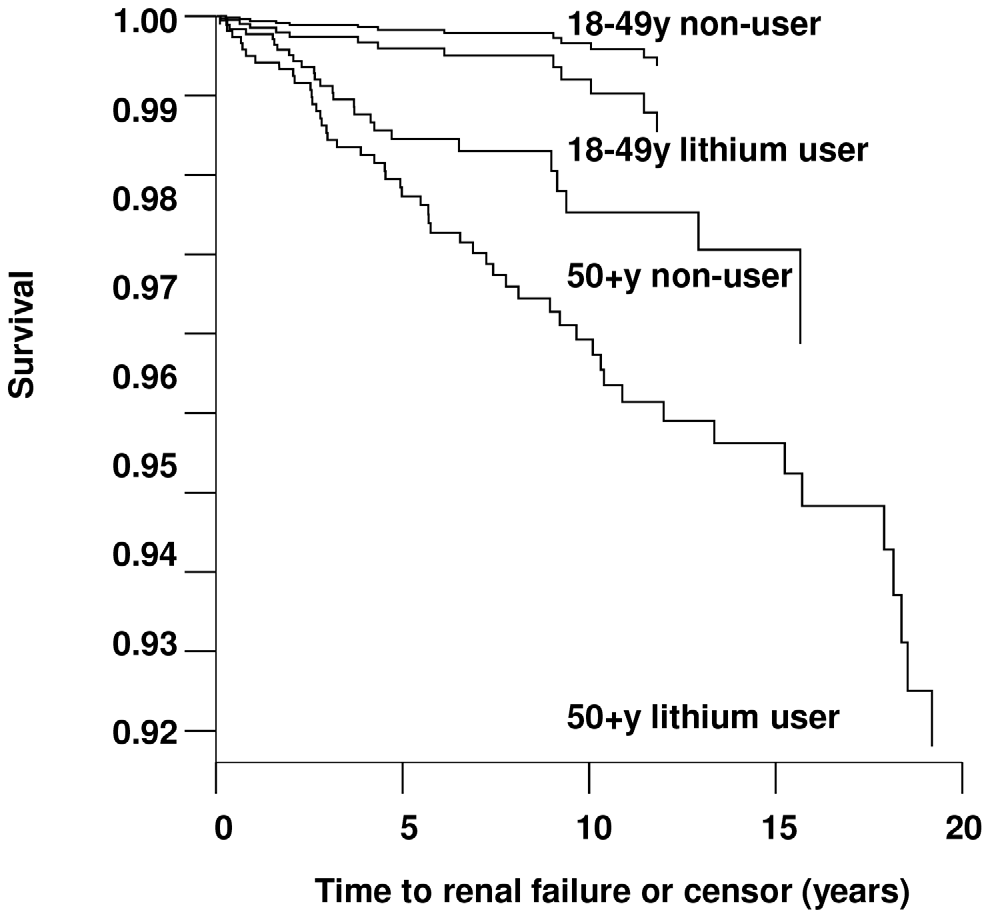
Renal failure survival curves for younger and older lithium and non-lithium users.

Fully adjusted multivariable analysis including the influence of covariates on renal failure is shown in Table 3. All covariates were initially included in the model, and final adjusted models demonstrated an association between renal failure and lithium use (HR: 2.7 (95% CI 1.7 to 4.3), diuretic use (HR: 2.3 (1.4 to 3.7), and diabetes (HR: 2.8 (1.6 to 4.6). Although diuretics showed a statistically significant association, hypertension as a stand-alone variable did not fit either model. Gender did not have a significant effect in any of the final adjusted model, nor did several traditional renal risk factors such as smoking and alcohol use. Long term NSAID use (≥30 days) was weakly associated with renal failure (HR 1.5 (0.7 to 3.2)) but was not statistically significant. The final models are consistent with age and gender adjusted models (Table 2). Given the effect of age on the models, data were partitioned into younger (18 to 49 years) and older (50+ years) cohorts and the model (fully adjusted, validated model shown in table 3) was re-evaluated. Although there was no interaction between age and lithium use within regression models in this study, increasing risk of renal failure with age meant that the absolute risk associated with lithium use in older patients was notable (Figure 2).

**Table 3:** Final adjusted hazard ratios for renal failure among lithium and non-lithium users (using validated data).

	*Unvalidated data* HR (95% CI), p	*Validated data* HR (95% CI), p
**Age**	1.04 (1.03 to 1.06), <0.001	1.04 (1.03 to 1.06), <0.001
**Lithium user**	2.4 (1.5 to 3.8), <0.001	2.7 (1.7 to 4.3), 0.001
**Diuretic**	2.3 (1.4 to 3.7), 0.001	2.3 (1.4 to 3.7), 0.001
**Diabetes**	2.8 (1.6 to 4.6), <0.001	2.7 (1.6 to 4.5), <0.001

### Model Specification

Reported models were tested for and rejected time dependency of covariates or interactions between variables in most instances. There were no instances of interaction between lithium use and other covariates. The findings for lithium are not confounded by age; reported models are adjusted by age as a continuous variable, but regression analyses using age as a categorical variable also show lithium exposure as an independent risk factor for renal failure.

### Cessation of Lithium and Persistence of Increased Risk

We examined whether lithium exposure was associated with a persistently increased risk of renal failure. Fifty-one lithium users developed renal failure during the evaluation period, and at least 35 of these renal failure events occurred during lithium treatment or within 6 months of cessation. The validation study (reported below) indicated an underestimation of lithium exposure duration; thus more refined exposure analyses were deemed unreliable and are not reported here.

### Full Text Validation Exercise and Sensitivity Analysis

Of 77 cases of renal failure, 44 (57%) records were available for extraction and analysis. Of the remainder, 3 died, 11 had changed to a different IT system preventing identification using the GPRD unique identifier, 4 patients had transferred out of the area with no notes available, 2 patients were with practices that no longer wished to participate in GPRD studies, and there were13 non-responses. Of the 44 patients whose records were analysed, all 28 patients previously identified as lithium users were confirmed. Lithium exposure duration was confirmed in all but 6 patients; of these, duration had potentially been under-estimated by between 4–32 years (mean 12.3 y, SD 10.3 y). There was insufficient information to assess whether exposure during these periods was continuous or interval. In the 16 patients previously identified as non-users of lithium, note review showed that 2 had evidence of lithium use in letters from secondary care (duration unknown). A sensitivity analysis was conducted taking into account the misclassified cases, with almost identical results.

Of the 44 patients whose records were analysed (and who were previously identified as having renal failure), we were able to confirm 33 (75%) cases of renal failure, of whom 3 had a record of dialysis. An additional 4 patients had a record of chronic kidney stage 3, and 3 had a record of renal impairment with stage unknown. Of the 33 confirmed cases of renal failure, the date of onset was available for 23 (70%); of these, 17 (74%) were within 30 days of the previously identified date, and the remainder of cases were within 1 year. A sensitivity analysis was conducted excluding un-validated cases of renal failure, again with almost identical results (Table 3 shows results from both unvalidated and validated data).

### Clinical Importance

Underlying risk of renal disease increases with age, consequently risk of harm was estimated for younger (<50 years) and older (≥50 years) cohorts of patients undergoing treatment. In patients under 50 years of age using lithium, the estimated absolute increase in risk of renal failure was 0.15% (number needed to harm, NNH: 660, 95%CI: 300 to 2100) and renal impairment was 0.95% (NNH: 105, 95%CI: 69 to 167). In patients over 50 years of age using lithium, the estimated increase in risk of renal failure was 2.3% (NNH: 44, 95%CI: 21 to 150) and renal impairment was 8.0% (NNH: 13, 95%CI: 8 to 20).

## Discussion

This retrospective cohort enquiry provides evidence for the strength of association linking lithium use with renal disease [31]. The linkage is further enhanced by consistency across renal outcome definitions (failure and impairment) and by insensitivity to adjustment for potential confounding variables. Lithium use was associated with a two and a half-fold increased risk in renal failure and nearly a three-fold increased risk of renal impairment. Our study confirms lithium’s role as an independent risk factor for renal failure, which increases with age.

The biological mechanism for renal impairment and renal failure in long-term lithium treatment remains to be fully elucidated [20], [32]. Although early biopsy studies found a range of renal pathologies in those exposed to lithium, these were not clearly linked to reduced glomerular function. More recent studies report a more consistent picture of tubulointerstitial disease in long term lithium users who develop renal impairment [33], with focal segmental glomerulosclerosis or multiple microcysts detectable by magnetic resonance imaging [34], which is likely to be due to a specific effect of lithium rather than the result of established common risk factors for renal failure [35]. It remains unknown whether specific risk factors exist which increase vulnerability to lithium-associated renal failure, and hence whether a high-risk group can be identified at the outset of treatment. There may be genetic factors, but a causal role for either acute or chronic lithium toxicity remains a plausible but untested hypothesis. It was not possible within the current study to explore the role of acute lithium poisoning which may have led to renal failure in this population. It remains uncertain to what extent renal damage occurs as a result of high serum lithium levels as opposed to individual susceptibility even at normal therapeutic serum levels of lithium. A recent systematic review [20] demonstrates that the majority of studies inconsistently report daily doses and duration of use, concurrent diabetes insipidus or acute renal injury, concomitant drug use, and exclude patients with acute lithium toxicity, thus the role of these risk factors remains uncertain. This study was unable to address these issues, and thus interpretation of findings is limited by the unknown influence of variations in dose, duration, toxicity, and interactions with other drugs.

Diagnostic criteria for the index diagnosis, bipolar disorder, have changed over time and GPs use different classifications for a variety of reasons, consequently psychiatry colleagues within the team developed a wide-ranging code list to ensure that we captured a wide cohort of patients with a diagnosis of bipolar disorder. The mean age of the cohort was slightly older than that reported in other studies of patients with bipolar disorder [36]; it is possible that GPRD may not record bipolar diagnosis until some years after a diagnosis is made in secondary care. It is also possible that patients treated with lithium represent more severe bipolar disorder and thus the findings may result from selection bias. However, findings show little difference between the lithium-exposed and non-exposed groups for a range of emergent disease parameters. It may be that the lithium-exposed group have more severe bipolar disorder, but they do not appear to have more severe physical disorders.

Cases of renal failure were selected using a highly specific code list, but the definition of renal impairment was wider; until recently (with the introduction of Quality Outcome Frameworks (QOF) in primary care [37]) GPs in England were not required to record the level of severity of renal disease. Possible inconsistencies in GP data entry using diagnostic codes may have led to an overestimation of the prevalence of renal impairment but underestimation of renal failure. Our validation study demonstrates reliable recording of renal failure and supports previous validation research [38]. However, it is notable that although the validated code list had high specificity, its sensitivity remains unknown and follow-up studies using laboratory confirmed renal failure (for example eGFR) are needed to confirm findings.

Previous research [22] identifies diabetes and hypertension as independent risk factors for renal disease. Within our study, diabetes and anti-hypertensive diuretic drug use were significantly associated with renal failure. However, in fully adjusted models, hypertension itself not associated with renal disease although this may be a result of historical GPRD data entry inconsistencies. NSAID use has also been shown in previous research [22] to be an independent explanatory variable for renal failure. In this study, long term NSAID was weakly associated with renal failure but was not statistically significant.

Prescriptions for lithium issued in secondary care, for example, in specialist psychiatric and nephrology clinics may not be recorded in the GPRD. Thus exposure to lithium may be underestimated in these patients. Our first reported validation study of lithium exposure supported GPRD dataset classification of exposure per se, but led us to conclude that lithium exposure duration determined using the GPRD dataset alone was unreliable, due to evidence of secondary care prescribing. Although most long-term prescribing for stable patients is likely to occur in primary care, drug therapy at onset or during crises may be managed by the secondary care psychiatrist. This is an example of ‘immeasurable time bias’, a recognized bias in pharmacoepidemiology studies resulting from missed exposure missed in observational databases due to hospital admission or outpatient secondary care [39]. It is possible that lithium use may be withdrawn due to development of renal impairment, while those on long-term lithium use may demonstrate a ‘healthy survivor’ effect. Patients exposed to lithium may have been offered more intensive screening, thus the study may be limited by ascertainment bias in that the unexposed group may have undetected renal disease. This may be more likely to occur in renal impairment. However, our primary outcome of renal failure represents more advanced disease, which will be less susceptible to this ascertainment bias.

Despite inevitable limitations of the retrospective cohort design this study provides consistent evidence of the link between lithium use and renal disease. In absolute terms there is a small increased risk of renal disease with lithium use. However this increases with age, with numbers needed to harm (NNH) for renal failure of 44 in patients aged 50 and over. There is a need for rigorous, routine renal monitoring from the onset of treatment, and for patients to be informed of this risk when deciding to use lithium therapy for bipolar disorder. The risks need to be balanced against the evidence for lithium’s comparative effectiveness in this group. Comprehensive examination of potentially important co-factors such as lithium exposure duration and lithium toxicity demand prospective studies in this and other diagnostic groups.

### Data Availability

GPRD Read code lists are available on request from h.j.close@durham.ac.uk.
